# Evaluation of *Pax6* Mutant Rat as a Model for Autism

**DOI:** 10.1371/journal.pone.0015500

**Published:** 2010-12-21

**Authors:** Toshiko Umeda, Noriko Takashima, Ryoko Nakagawa, Motoko Maekawa, Shiro Ikegami, Takeo Yoshikawa, Kazuto Kobayashi, Kazuo Okanoya, Kaoru Inokuchi, Noriko Osumi

**Affiliations:** 1 Division of Developmental Neuroscience, Tohoku University Graduate School of Medicine, Sendai, Japan; 2 Laboratory for Behavioral and Developmental Disorders, RIKEN Brain Science Institute, Wako, Japan; 3 Mitsubishi Kagaku Institute of Life Sciences (MITILS), Tokyo, Japan; 4 Laboratory for Biolinguistics, RIKEN Brain Science Institute, Wako, Japan; 5 Laboratory for Molecular Psychiatry, RIKEN Brain Science Institute, Wako, Japan; 6 Department of Psychology, Saitama Institute of Technology, Fukaya, Japan; 7 Department of Molecular Genetics, Institute of Biomedical Sciences, Fukushima Medical University School of Medicine, Fukushima, Japan; 8 Department of Biochemistry, Faculty of Medicine, Graduate School of Medicine and Pharmaceutical Sciences, University of Toyama, Toyama, Japan; Tokyo Institute of Psychiatry, Japan

## Abstract

Autism is a highly variable brain developmental disorder and has a strong genetic basis. Pax6 is a pivotal player in brain development and maintenance. It is expressed in embryonic and adult neural stem cells, in astrocytes in the entire central nervous system, and in neurons in the olfactory bulb, amygdala, thalamus, and cerebellum, functioning in highly context-dependent manners. We have recently reported that *Pax6* heterozygous mutant (*rSey^2^*/+) rats with a spontaneous mutation in the *Pax6* gene, show impaired prepulse inhibition (PPI). In the present study, we further examined behaviors of *rSey^2^*/+ rats and revealed that they exhibited abnormality in social interaction (more aggression and withdrawal) in addition to impairment in rearing activity and in fear-conditioned memory. Ultrasonic vocalization (USV) in *rSey^2^*+ rat pups was normal in male but abnormal in female. Moreover, treatment with clozapine successfully recovered the defects in sensorimotor gating function, but not in fear-conditioned memory. Taken together with our prior human genetic data and results in other literatures, *rSey^2^*/+ rats likely have some phenotypic components of autism.

## Introduction

Autism is a highly variable developmental brain disorder defined by three core symptoms with onset prior to 3 years of age: atypical social behavior; disrupted verbal and non-verbal communication; and unusual patterns of highly restricted interests and repetitive behaviors [1]–[4]. In addition to the major symptoms, there are several associated emotional manifestations in autism, such as depression and increased anxiety and fear [5]–[7]. Furthermore, the concept of autism itself has been broadened and now includes the group of syndromes referred to as pervasive developmental disorders including Asperger's disorder, pervasive developmental disorder not otherwise specified, childhood disintegrative disorder, and Rett's disorder in the *Diagnostic and Statistical Manual of Mental Disorders IV* [4]. Collectively, these disorders are known as the autism spectrum disorders (ASD). The existence of multiple symptom domains and the spectrum of related disorders demonstrate the complexity of the autism phenotype.

Several twin studies indicate that concordance rates for monozygotic twins (70–90%) are several-fold higher than those for dizygotic twins (−10%) [2], [8]–[10]. These indicate that genetic factors play important roles in its etiology. However, the identification of susceptibility genes has been hindered by the heterogeneity of the syndrome, insufficient numbers of analyzed samples and small effect size of each risk gene, compared to other physical complex disorders [11]. Under these circumstances, to search for rare risk variants with substantial effects may be a fruitful approach [12].

Autism is psychiatric illnesses in which complicated information processing might be disturbed at different levels of brain development, introducing substantial heterogeneity [13]. Fundamental differences in the underlying neurodevelopmental disruptions probably lead to the heterogeneity in both symptoms and developmental course that are characteristic of autism. Various genes operate to form the brain through neurogenesis, gliogenesis, area formation and neuronal circuit formation. *Pax6* gene encodes a transcription factor that is essential for neurodevelopment, and is expressed in restricted regions of the forebrain, hindbrain, and spinal cord appearing as early as at embryonic day 8.5 (E8.5) and throughout life in certain brain regions such as the amygdala, olfactory bulb, pyriform cortex, and dentate gyrus and also in astrocytes [14]–[17]. Human *PAX6* gene is originally identified in chromosomal region 11p13 as one related with WAGR (Wilm's tumor, Aniridia, Genitourinary malformations and mental Retardation) syndrome [18], [19], which is a rare genetic disorder caused by chromosomal deletion of the 11p12-p14 region. The majority of WAGR patients have mental retardation and behavioral problems, and importantly, more than 20% of the patients also have features of autism [20], [21]. Recent studies have identified *PAX6* mutations in individuals who manifest mental retardation, aniridia and autism [22]–[24]. Furthermore, chromosome 11p13, on which *PAX6* is located, is implicated as a possible locus for autism susceptibility by a linkage study [25]. These accumulating lines of evidence suggest that *PAX6* mutations display concomitant phenotypes of autism. Recently, our group has carried out resequencing analysis of the gene in autistic patients with aniridia, with the aim of searching for additional mutations of *PAX6*, which might be associated with the disease. As a result we have detected a novel missense mutation of *PAX6* in an autistic patient, not found in 2,120 non-autistic subjects [26]. These suggest that a part of autistic patients carry rare *PAX6* mutations and that *Pax6* dysfunction during neurodevelopment might be responsible for autistic behaviors.

To address this issue using experimental animals in the current study, we performed the detailed analyses of *rSey^2^*/+ rats that have spontaneous nonsense mutations in the *Pax6* gene [27], [28], in terms of behavioral tests, biochemical analysis and pharmacological examination.

## Results

### Abnormal exploratory behavior toward a novel environment in *rSey^2^/+* rats

*rSey^2^/+* rats were fertile and showed no change in sexual behavior and litter size compared with the wild type rats (WT). Apparently, *rSey^2^/+* rats exhibited no ataxia or seizure, and moved normally as judged from their footprint patterns (data not shown). To assess whether *rSey^2^/+* rats display autistic phenotypes, we executed several behavioral testes using only male rats to avoid potential influence of menstrual cycles except for USV test.

**Figure 1:**
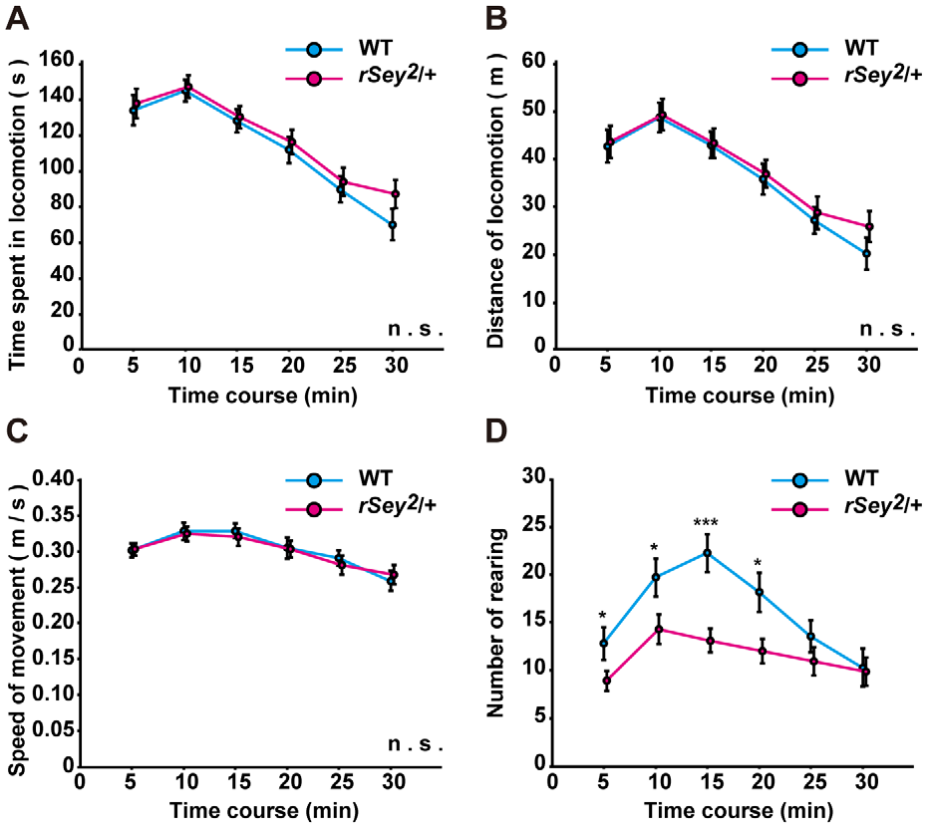
General motor activity of WT and *rSey^2^/+* rats in an open-field test. Time spent in locomotion (**A**), distance of locomotion (**B**), speed of movement (**C**), rearing counts (**D**) were measured in WT (blue, n = 22) and *rSey^2^/+* rats (magenta, n = 28). *rSey^2^/+* rats exhibited decrease in the number of rearing compared to WT (genotype: F(1) = 6.54, P<0.02; time: F(5) = 17.13, P<0.001; genotype × time: F(5) = 3.74, P<0.005; **D**), although time spent in locomotion (**A**), distance of locomotion (**B**), and speed of movement (**C**) were not significantly changed. Data are expressed by mean ± SEM. n.s., not significant ***P<0.001, compared to WT as determined by Bonferroni post hoc test.

Firstly we conducted open-field tests using a box equipped with photobeam sensors given a 15 minutes session [29], [30]. In this test, no significant differences were observed in times spent in locomotion, distances of locomotion, speed of movement and times spent in the central and peripheral zone of the open field (**Fig. 1A–C** **, data not shown**). However rearing activity was significantly lower in *rSey^2^/+* rats than in WT (two-way analysis of variance (ANOVA) with repeated measures (genotype × time); main effect of factor ‘genotype’: F(1) = 6.54, P<0.02; **Fig. 1D**). The result suggests that *rSey^2^/+* rats may be less exploratory or more anxious to their novel environment [31], [32].

**Figure 2:**
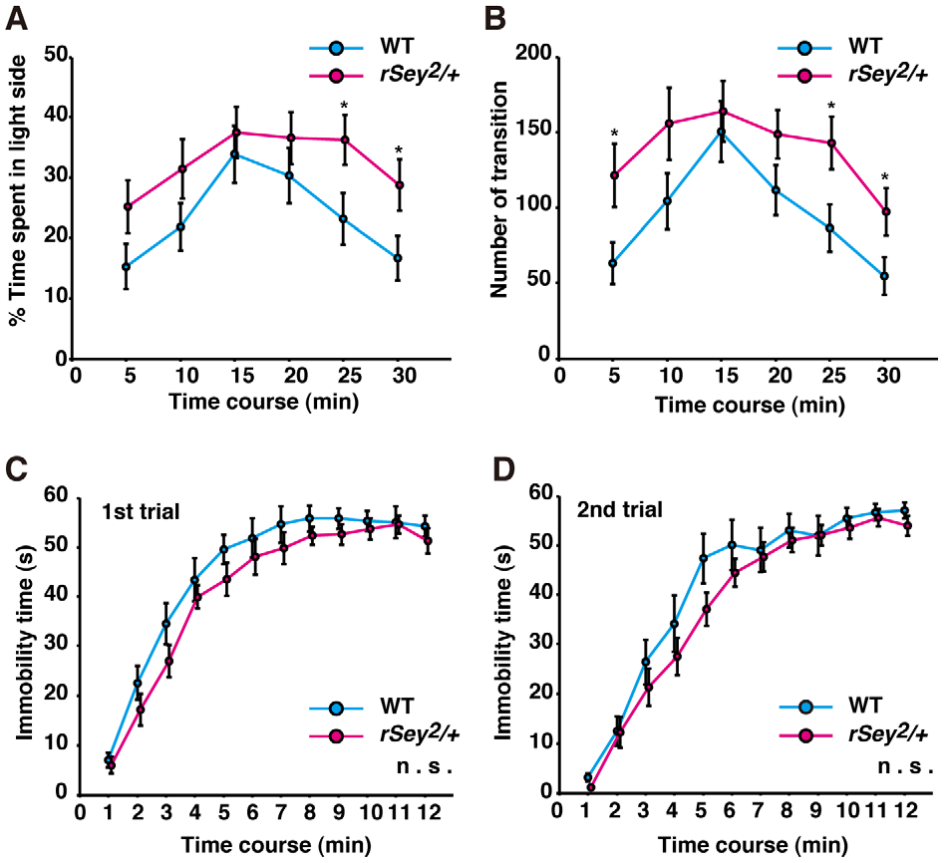
Anxious and depressive-like behavior in WT and *rSey^2^/+* rats. Anxious-like behavior was evaluated using a LD test. The mean percentage of time spent in the light compartment (**A**) and the number of transitions between the two compartments (**B**) were measured in WT (blue, n = 23) and *rSey^2^/+* rats (magenta, n = 28). *rSey^2^/+* rats spent longer time in the light compartment (genotype: F(1) = 7.06, P<0.02; time: F(3.86) = 6.91, P<0.001: genotype × time: F(3.86) = 0.56, not significant; **A**) and exhibited more number of transitions between the two compartments (genotype: F(1) = 6.02, P<0.02; time: F(3.41) = 5.56, P<0.001; genotype × time: F(3.41) = 0.49, not significant; **B**), compared to WT. Depressive-like behavior was evaluated using a forced swim test. Immobility time was calculated in first trial of test (**C**) and in second trial performed 24 h after the first trial (**D**). Immobility times were not changed between WT (blue, n = 11) and *rSey^2^/+* rats (magenta, n = 16). Data are expressed by mean ± SEM. n.s., not significant ***P<0.001, compared to WT as determined by Bonferroni post hoc test.

To assess the level of anxiety in *rSey^2^/+* rats, we next carried out a light-dark (LD) choice test [33]. *rSey^2^/+* rats spent more time in the light side of the test box than that WT did (two-way ANOVA with repeated measures (genotype × time); main effect of factor ‘genotype’: F(1) = 7.06, P<0.02; **Fig. 2A**). In addition, the number of entries into the dark box was increased in *rSey^2^/+* rats compared with that of WT (two-way ANOVA with repeated measures (genotype × time); main effect of factor ‘genotype’: F(1) = 6.02, P<0.02; **Fig. 2B**). Because rodents prefer the dark environment, *rSey^2^/+* rats might be less anxious. However, we have to bear in mind that *rSey^2^/+* rats have eye defects such as decreased eye size, and various levels of cataract and iris hyperplasia, though these phenotypic variation seems to be less than those in *Sey* mice [34]. *rSey^2^/+* rats could discriminate a difference between lightness and darkness since the time percentages spent in the light and dark sides were not equal. Anyway, the results of LD test should be interpreted with caution.

To further examine emotional behavior in *rSey^2^/+* rats, we performed forced swim test [35]. There were no differences between WT and *rSey^2^/+* rats in immobility times at 1st and 2nd trials of the tests (**Fig. 2C, D**). Taken altogether, it is reasonable to assume that impaired rearing activity in *rSey^2^/+* rats may not be related to increased anxiety or depressed state but rather to a less exploratory phenotype.

### Abnormality in social interactions in *rSey^2^*/+ rats

A critical component in a model animal of autism is a quantitative measure of appropriate social interaction. To address this issue, we examined social interactions of WT and *rSey^2^/+* rats by measuring time spent in locomotion, aggression, following, passive body contact, allo-grooming, mounting, sniffing, and isolation (WT, 14 pairs; *rSey^2^/+* rats, 16 pairs).

**Figure 3:**
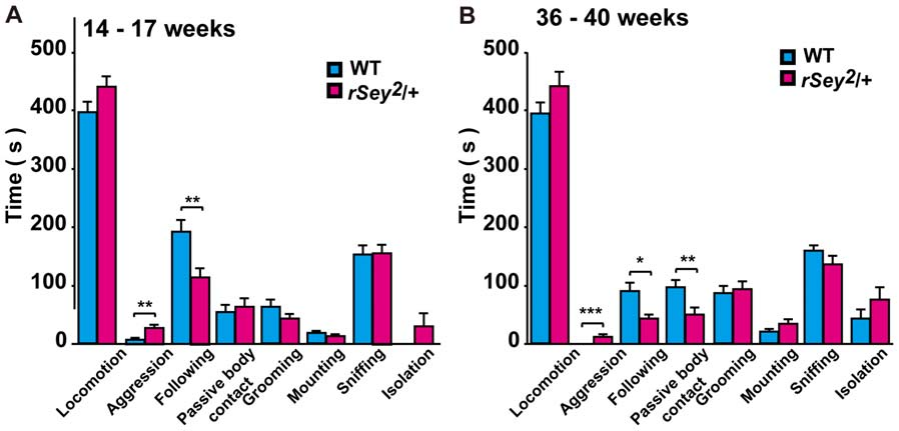
Social interaction test in WT and *rSey^2^/+* rats. Time spent for social behaviors, including locomotion, aggression, following, passive body contact, allo-grooming, mounting, sniffing and isolation were measured in WT and *rSey^2^/+* rats using an open-field box (**A, B**). A pair of WT (blue, 14 pairs) or a pair of *rSey^2^/+* rats (magenta, 16 pairs) at 14–17 weeks old was placed in an open-field box. *rSey^2^/+* rats exhibited more aggressive behavior and less following compared with WT (**A**). A pair of WT (blue, 12 pairs) or a pair of *rSey^2^/+* rats (magenta, 13 pairs) at 36–40 weeks old also exhibited more aggressive behavior but less following. In addition, decreased passive body contact was observed (**B**). Data are expressed by mean ± SEM. *P<0.05, **P<0.01, ***P<0.001, compared to WT as determined by *t*-test.

At 14–17 week-old, *rSey^2^/+* rats clearly showed more aggressive behavior than WT (*t*-test; P<0.01; **Fig. 3A**), while the former showed less following behavior than the latter (*t*-test; P<0.01; **Fig. 3A**). There were no differences in the other behaviors (locomotion, passive body contact, allo-grooming, mounting, sniffing and isolation). Interestingly, these features became much more obvious at full adult stages (36–40 weeks). *rSey^2^/+* rats of these ages still showed aggressive behavior, although aged WT rats never did (*t*-test; P<0.001; **Fig. 3B**). Moreover, *rSey^2^/+* rats exhibited significantly less following (*t*-test; P<0.05; **Fig. 3B**) and passive body contact behaviors (*t*-test; P<0.01; **Fig. 3B**). Thus *rSey^2^/+* rats clearly exhibited abnormalities in social interaction; they were more aggressive at fighting and less interested in a strange partner.

### Abnormality in fear-conditioned memory in *rSey^2^/+* rats

**Figure 4:**
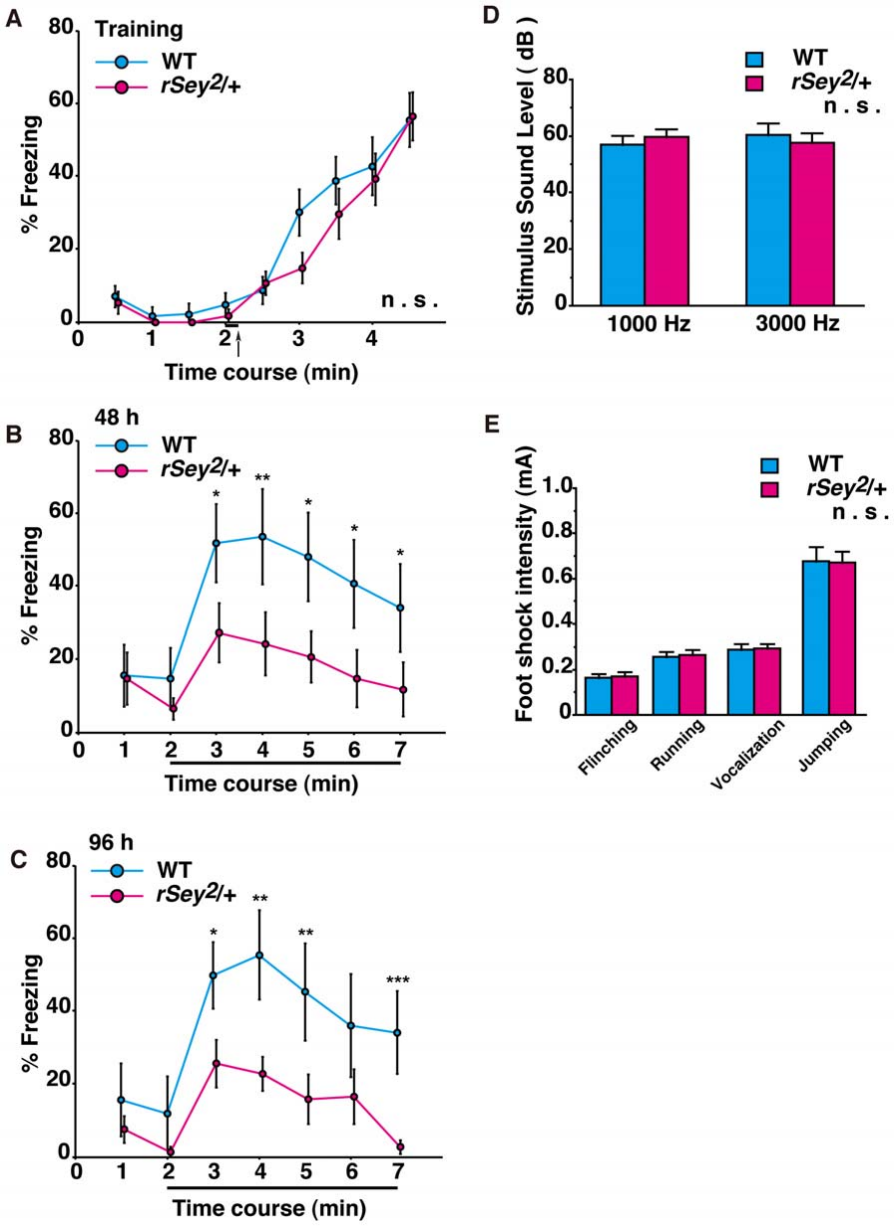
Tone-fear conditioned memory in WT and *rSey^2^/+* rats. For training, each rat was placed in the test chamber for 2 min, and subsequently received a pair of tone (20 s, horizontal bar) and foot shock (0.3 mA, arrow) (**A**). The mean percentage of time spent freezing was plotted in WT (blue, n = 22) and *rSey^2^/+* rats (magenta, n = 28). Forty-eight h (**B**) and 96 h (**C**) after the training, freezing responses to the tone were measured for 5 min (horizontal bars) in a context-independent way. Freezing response was not different between WT (n = 22) and *rSey^2^/+* rats (n = 28) during the initial training (**A**). In contrast, performance of tone-fear conditioned memory was lower after 48 h (genotype: F(1) = 9.20, P<0.01; time: F(2.35) = 5.92, P<0.005; genotype × time: F(2.35) = 0.25, not significant; **B**) and 96 h (genotype: F(1) = 11.40, P<0.005; time: F(2.62) = 7.96, P<0.001; genotype × time: F(2.62) = 0.99, not significant; **C**) in *rSey^2^/+* rats. Auditory ability (**D**) and sensitivity to electric foot shock (**E**) in *rSey^2^/+* rats were comparable to that in WT. Data are expressed by mean ± SEM. n.s., not significant **P<0.01, ***P<0.001, compared to WT as determined by Bonferroni post hoc test.

Next we examined performance in memory of *rSey^2^/+* rats. Because behavioral tasks requiring visual function may be influenced by potential visual impairment in *rSey^2^/+* rats due to eye abnormalities, we chose a tone fear-conditioning test using acoustic stimuli in a context-independent way [36]. For conditioning, an electrical shock (0.3 mA) was given just after 20 s tone (**Fig. 4A**). Although freezing response immediately after the foot shock (**Fig. 4A**) was not different between WT and *rSey^2^/+* rats, the latter exhibited significantly reduced freezing response 48 h (two-way ANOVA with repeated measures (genotype × time); main effect of factor ‘genotype’: F(1) = 9.20, P<0.01; **Fig. 4B**), and 96 h (two-way ANOVA with repeated measures (genotype × time); main effect of factor ‘genotype’: F(1) = 11.40, P<0.005; **Fig. 4C**) after the initial training session. Since auditory ability and electric foot shock sensitivity may affect freezing responses, we performed auditory threshold test (**Fig. 4D**) and shock sensitivity test (**Fig. 4E**). There were no differences in auditory threshold for orienting sound source and shock sensitivity. Therefore, *rSey^2^/+* rats had low performance in the context-independent tone-fear conditioned memory.

### Abnormality in ultrasonic vocalization test

**Figure 5:**
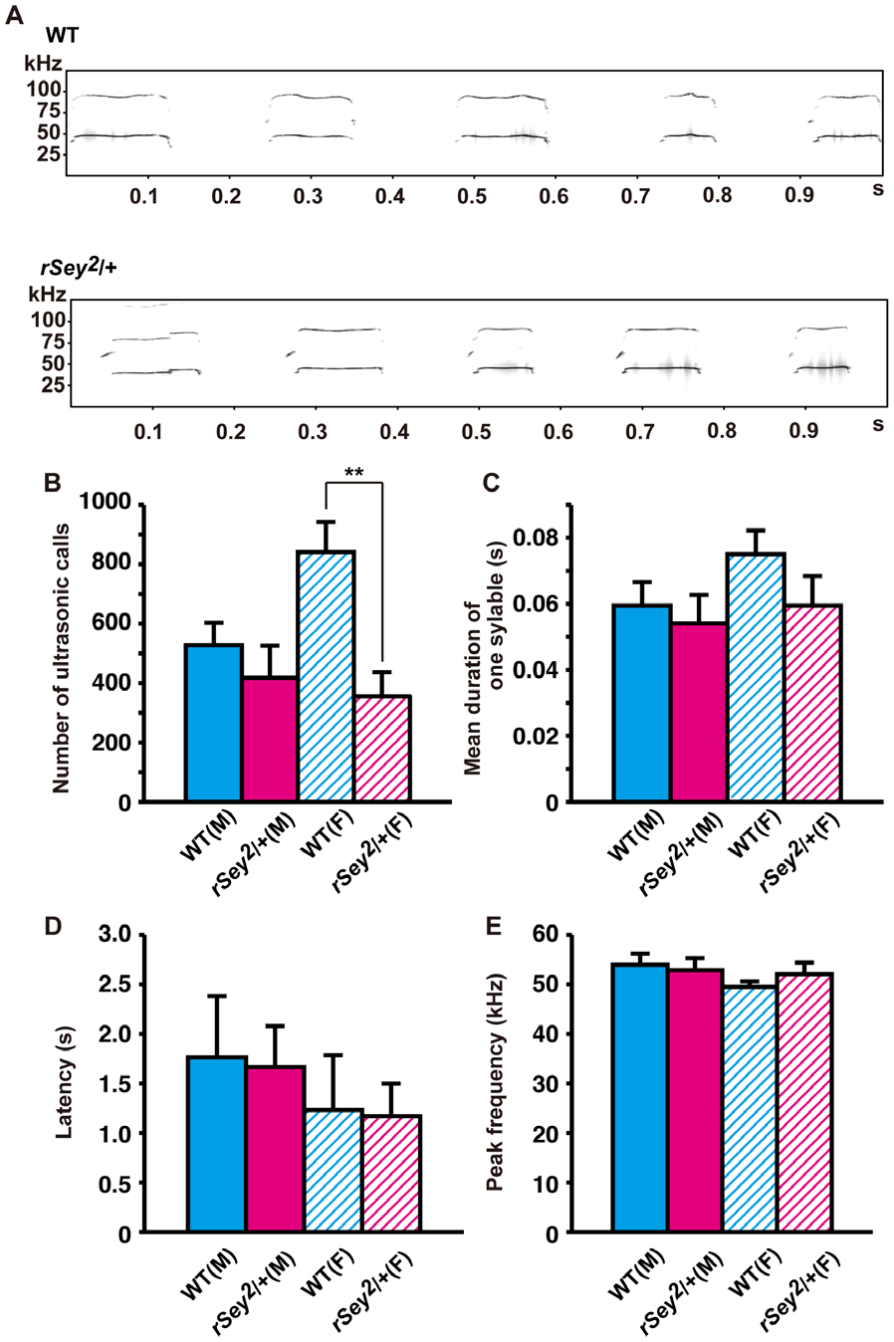
Ultrasonic vocalization in WT and *rSey^2^/+* rat pups. The USV of individual rat pups (WT male: n = 18; WT female: n = 9; *rSey^2^/+* male: n = 11; *rSey^2^/+* female: n = 13) were measured during the isolation condition on P7. Spectrograms (frequency, kHz × time, s) of USV produced by WT (*Upper*) and *rSey^2^/+* male pups (*Lower*) (**A**). Column graphs comparing among WT male (closed blue line), *rSey^2^/+* male (closed magenta line), WT female (hatched blue line) and *rSey^2^/+* female (hatched magenta line) pups in the number of calls per trial (**B**), mean duration of one call (s) (**C**), latency of the first vocalization (s) (**D**), and the peak frequency (kHz) (**E**). *rSey^2^/+* female rats exhibited decrease in the number of calls compared to WT female rats (genotype: F(1) = 10.53, P<0.002; gender: F(1) = 1.87, not significant; genotype × gender: F(1) = 4.20, P<0.05; **B**). Data are expressed by mean ± SEM. **P<0.01, compared to WT female rats as determined by Bonferroni post hoc test.

Next we tested communicative behavior of *rSey^2^/+* rats by recording isolation-induced USV [37] of male postnatal day 7 (P7) rat pups that were separated from their dams. Number of ultrasonic calls (**Fig. 5A**), and mean duration of calls (**Fig. 5B**), latency to start calling (**Fig. 5C**), and peak frequency (**Fig. 5D**) were not different between WT and *rSey^2^/+* male rat pups, suggesting that communicative behavior of *rSey^2^/+* male rats was normal. We also conducted USV test on *rSey^2^/+* female rat pups since they were not yet influenced by sexual cycles. It is of note that *rSey^2^/+* female rat pups emitted fewer calls than WT (two-way ANOVA measures (genotype × gender); interaction: F(1) = 4.20, P<0.05; main effect of factor ‘genotype’: F(1) = 10.53, P<0.002; not significant main effect of factor ‘gender’; **Fig. 5B**). These results imply that *rSey^2^/+* rat female pups exhibit less property toward their mothers as similarly observed in autistic infants. This finding is quite interesting since we reported an autistic girl who has a mutation in the *PAX6* gene inherited from her father who is not diagnosed as autism [26].

### Decreased serotonin levels in plasma and brain samples of *rSey^2^/+* rats

**Figure 6:**
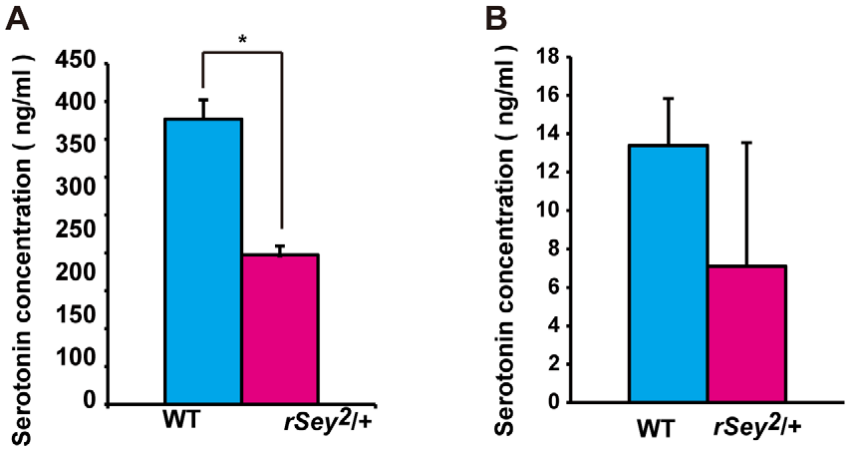
Reduced serotonin concentration in *rSey^2^/+* rats. Contents of 5-HT in PPP (**A**) and the hippocampus (**B**) were analyzed by using HPLC equipped with an electrochemical detection system. 5-HT levels in PPP of *rSey^2^/+* rats (magenta, n = 8) were lower than in WT (blue, n = 9) (*t*-test; P<0.05, **A**), while 5-HT level in the hippocampus of *rSey^2^/+* rats (magenta, n = 5) was marginally reduced compared to that in WT (blue, n = 6) (*t*-test; P = 0.83, **B**). Data are expressed by mean ± SEM. n.s., not significant *P<0.05, compared to WT as determined by *t*-test.

Although the relationship between autism and abnormal serotonin (5-HT) levels is still unclear, an imbalance of 5-HT concentrations in brain and/or in blood is believed to cause many of the characteristic symptoms of autism [38]–[40]. We measured platelet-poor-plasma (PPP) 5-HT levels in adult male rats (18–20 weeks) using high-performance liquid chromatography (HPLC) equipped with an electrochemical detection system. As shown in **Fig. 6A**, PPP 5-HT levels in *rSey^2^/+* male rats were lower than in WT (*t*-test: P<0.05). We also measured the 5-HT level in the hippocampus. Intriguingly, 5-HT level in the *rSey^2^/+* hippocampus was marginally reduced compared to that in WT (*t*-test: P = 0.083; **Fig. 6B**).

### Anti-psychotic drug recovers PPI

**Figure 7:**
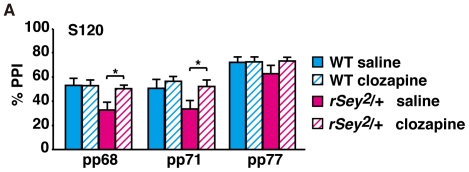
Clozapine recovers PPI defect in *rSey^2^/+* rats. Both WT and *rSey^2^/+* rats were injected intrapenitorially either with saline (WT: closed blue line, n = 11; *rSey^2^/+* rat: closed magenta line, n = 11, **A**) or clozapine (WT: hatched blue line, n = 11; *rSey^2^/+* rat: hatched magenta line, n = 11, 1.5 mg/kg body weight, hatched line, **A**) 30 min prior to each test. Clozapine treatment specifically recovered the defect in PPI with 120 dB startle stimulus (*t*-test; P<0.05, **A**). Data are expressed by mean ± SEM. *P<0.05, compared to WT as determined by *t*-test.

One goal to establish a rodent model for mental diseases is to use it for discovery of new drugs for therapy. As described above, *rSey^2^/+* rats showed several phenotypes related to autism together with impairment of PPI [41]. In addition, 5-HT levels were abnormal in the brain and serum of *rSey^2^/+* rats. Therefore, we tested effects of clozapine, an anti-psychotic drug that targets both 5-HT receptor and dopamine receptor D_4_ [42]. WT and *rSey^2^/+* rats at 12–16 weeks were intraperitoneally treated either with saline (control) or with clozapine (1.5 mg/kg body weight) 30 min before behavior tests. Interestingly, *rSey^2^/+* rats treated with clozapine improved scores of PPI (*t*-test: P<0.05; **Fig. 7A**). In marked contrast, clozapine-treated *rSey^2^/+* rats showed no recovery on rearing behavior and on tone-fear conditioned memory at 48 h and 96 h (data not shown). Moreover, clozapine had no effects on foot shock sensitivity and acoustic startle response (data not shown). These results suggest that clozapine is effective to improve sensorimotor deficits in *rSey^2^/+* rats but does not alter other behavioral phenotypes.

## Discussion

In this study, we examined whether and/or how much *Pax6* heterozygous rats model autism. *Pax6* homozygous mutant mice/rats, in which Pax6 functions are completely lost, die at birth with severe defects in the formation of the eyes, nose, forebrain, and spinal cord [see review by [43]]. There is a case report describing that a fetus with compound homozygous mutations in *PAX6* gene exhibits similar congenital defects [44]. On the other hand, *Pax6* heterozygous mice/rats are viable and fertile, and show slight defects in formation of the eyes, olfactory bulb and cerebrum [34], [45]–[47]. In human, haploinsufficiency of *PAX6* causes the absence or hypoplasia of the anterior commissure, decreased volumes of the corpus callosum and smaller brain size, in addition to aniridia and various eye abnormalities [48]–[52]. In addition, 11p12-13 locus covering *PAX6* gene is suggested as one of the autism linkage loci [25], and we have previously reported a mutation in *PAX6* found in a autistic patient [26]. These data suggest that *PAX6* haploinsufficiency may give rise to subtle abnormality in brain structures, which may lead to developmental disorders such as autism. Our present animal study revealed that *rSey^2^/+* rats indeed displayed some behavioral abnormalities and 5-HT system deficits related to autism.

### Comparison of phenotypes between *rSey^2^/+* rats and conditional *Pax6* KO mice

In a recent study, conditional *Pax6* knockout mice (*Pax6^fl/fl^; Emx1-Cre* mutants; *Pax6* cKO mice) in which *Pax6* expression was completely and specifically abolished in the cortex and hippocampus were generated [53]. They exhibit drastically severe cortical deficits such as loss of upper layers and prefrontal cortex markers, which are not seen in human autism. These *Pax6* cKO mice demonstrated a decreased locomotor activity and ataxia due to defects in motor performance and prefrontal deficits, whereas locomotor activity of *rSey^2^/+* rats was normal. Intriguingly, phenotypes of recent memory and extinction of cued fear that are related to amygdala functions, are different between *Pax6* cKO mice and *rSey^2^/+* rats. It is considered that Pax6 function may be normal in the *Pax6* cKO amygdala because promoter activity of *Emx1* is not working in this brain area [54], whereas expression levels of *Pax6* in the amygdala are deemed to be decreased in *rSey^2^/+* rats. As expected, this amygdala-dependent cued-fear conditioned recent memory is normal in *Pax6* cKO mice but impaired in *rSey^2^/+* rats.

PPI that reflects the sensorimotor gating system is reported to be abnormal across neuropsychiatric disorders including autism [55] and Asperger's disorder [56]. PPI scores were not changed in both *Pax6* cKO mice and *rSey^2^/+* rats at juvenile. Interestingly, *rSey^2^/+* rats exhibited decreased PPI at 12 weeks and afterwards [41]. These results may mean that impaired Pax6 functions could elicit altered inhibitory control of sensory input which is not obvious in younger animals.

### Sex differences in impairment of USV in *rSey^2^/+* rats

Abnormal reciprocal social interactions and communication deficits are the two of the three diagnostic symptoms of autism [57]. One well-characterized method for apparent social communicative interactions in rodents is the USV emitted by pups when they are out of the nest. Low levels of this type of infant vocalization may be relevant to the statements by some parents that their autistic children seldom cried and were easy to raise [58]. In literature, several autism models with mutations in *Foxp2*, *Nlgn4*, and *Tsc2* exhibit reduced USV [59]–[61]. Here we detected unusual properties of vocalizations in only female *rSey^2^/+* rat pups. These results might suggest that female *rSey^2^/+* rats exhibit impaired communication, which is the second core symptom of autism. Clinical and epidemiological studies on autism indicate that the disease incidence is higher in boys than in girls (ratio 4∶1), although the reason is not clear [see review by [62]]. It is known that girls with autism evidence greater communication deficits than boys, while boy sufferers show more restricted, repetitive, and stereotyped behaviors than girls [62], [63]. Hence, it seems that there are sex differences in pathogenic mechanisms. It is of note that there are sex-dependent differences in the hippocampal 5-HT transporter levels and dendritic spine densities [64]–[66]. Further work is needed to reveal detailed pathogenic mechanisms of sex-dependent phenotypic differences seen in *rSey^2^/+* rats.

### Altered 5-HT levels and PPI in *rSey^2^/+* rats

Several studies have demonstrated high levels of 5-HT in whole blood or platelets in approximately 25–30% of autistic patients [see review by [67]]. Given that 99% of circulatory 5-HT are accumulated in platelets, the measurement of platelet-poor plasma (PPP) 5-HT is essential for the evaluation of ‘free’ 5-HT, since this may reflect amount of brain synaptic 5-HT. In the present study, we found decreased 5-HT levels both in PPP and in the hippocampus of *rSey^2^*/+ rats, representing the first rodent model that shows impaired 5-HT conditions. Although there are few reports on PPP 5-HT levels in autistic adults, one paper has reported that lower PPP 5-HT levels might relate to the pathophysiology and symptomatology of autism [68].

Pharmacological studies suggest differential roles of 5-HT receptor subtypes in the modulation of PPI [69], [70]. 5-HT_2A_ receptor (HTR2A) is widely expressed in the central nervous system [71]. Elevated HTR2A activation by 5-HT could potently attenuate PPI [72]. Interestingly, we showed recovery of impaired PPI in *rSey^2^/+* rats using acute administration of clozapine, an anti-psychotic drug that shows strong binding to HTR2A [73] and decreases *HTR2A* mRNA [74]. We consider that HTR2A expression might increase in compensation to decreased 5-HT levels in synaptic clefts, which might induce impaired PPI in *rSey^2^/+* rats.

In summary, *rSey^2^/+* rats may potentially be used for discovery of effective drugs for autism, because these animals mimic at least some phenotypes of the disease.

## Methods

### Animals

Large colonies of *rSey^2^/+* rats and wild type Sprague-Dawley (SD) rats (littermates of *rSey^2^/+* rats) were maintained in Tohoku University Graduate School of Medicine and in Yamanouchi Pharmaceutical Company. All rats were maintained with a 12/12 light cycle (lights on at 8∶00 am) under temperature (22–24°C) and humidity (50–60%) controlled conditions. Food and water were available ad libitum. All the behavioral tests were conducted between 13∶00 and 18∶00 h. One week before the beginning of behavioral tests, the rats were housed one per cage and were handled once a day for 5 days. Genotype of *rSey^2^/+* rats was externally distinguishable because they have eye defects [27]. Age-matched male rats of littermates at various postnatal stages were used in this study. All the animal experiments were carried out in accordance with the National Institute of Health guidance for the care and use of laboratory animals and were approved by The Committees for Animal Experiments in Tohoku University Graduate School of Medicine (21–252), Mitsubishi Kagaku Institute of Life Sciences (MITILS-00-003) and RIKEN (H19-2B109).

### General activity test

General activity was measured in an open-field box (80×80×40 cm) made of gray vinyl chloride plates as described previously [30]. The apparatus was placed in a sound-attenuating room where external noise was greatly reduced (−45 dB at 500 Hz). Two pairs of 7×7 array infrared photosensors were attached to the outer wall equally spaced in lower and upper rows at intervals of 2 cm and 4.5 cm above the floor. The lower row of photocells was used to measure locomotor activity and the upper row to detect rearing behavior. A computer recorded the number of horizontal photobeam interruptions caused by animal movement. Each rat at 12–15 weeks old was placed into the apparatus and remained for 30 min.

### Social behavior recording

A pair of WT or a pair of *rSey^2^/+* rats were placed in an open-field box (80×80×40 cm) and behavior of the two rats were video recorded for 15 min. Recorded behavior was analyzed by measuring periods for various types of behavior. The aggressive behaviors (aggression) were kicking, wrestling, and defeating. Locomotion and isolation indicate that both animals behaved independently with movement and rest, respectively. Passive body contact indicates that both animals rested in contact with each other. The other behaviors were following, allo-grooming, mounting, and sniffing the partner. The scoring was carried out by an observer blind to the genotype. A blind observer manually measured time spent in each categorized behavior.

### Light–dark choice test

The apparatus consisted of two compartments made of gray vinyl choloride plates and placed in a darkened and sound-attenuating room. One compartment was a bright (250 lux) chamber (40×80×40 cm) illuminated by a white bulb (100 W) and the other was a dark (0.5 lux) chamber (40×80×40 cm). The two compartments were separated by a wall and connected by a small opening (8×20 cm) through which the photobeams of the sensors passed. A rat at 13–16 weeks old was placed in the center of the light chamber, and its behavior was recorded for 30 min. The rat was considered to have entered a new area when all four feet were in this area. The following behavioral measures were scored: the times spent in the light and dark compartments, the number of transitions between the two compartments, and the latency of the initial movement from the light to the dark room.

### Forced swim test

Forced swim test was performed as described [75]. On the first day of the test, each rat at 17–20 weeks old was placed and kept in the water for 12 min. The test was carried out in a cylindrical plexiglass tank (40 cm high and 30 cm in diameter), filled with water (25±1°C) up to a level of 20 cm. On the second day, 24 h later, the rat was placed in the tank for 12 min. After each swim session, the rat was dried gently with paper towel and returned to its home cage. These trials were videotaped for later analysis. The rat's movement during the swim test was measured using an infrared sensor system with a multi-Fresnel lens (CompACT FSS system, Muromachi, Tokyo, Japan) which was placed 20 cm directly above the surface of the water. The sensor monitors movement by detecting any object with a temperature 5°C higher than background within the tank. Behavior in the tank was scored every second as either swimming or immobility by a computer on the criteria of movement counts detected by the sensor. Immobility was defined as less than one movement count per second, which was approximately the same level that a trained observer judged as immobility while viewing the videotape. The time spent in immobility was calculated every minute.

### Tone-fear conditioning test

Fear conditioning was carried out as described [30] with minor modifications. On the training day, each rat was placed in a triangular prismatic chamber (SGS 002, Muromachi, Tokyo, Japan) for 2 min, and given a single conditioning trial consisting of a 20 s of a 65 dB tone at 1,000 Hz (conditioned stimulus) that ended at the same time as a 0.3 mA, 1 s-foot shock (unconditioned stimulus). The trained rats at 16–20 weeks old were removed from the chamber 2 min after the foot shock and returned to their home cages. Forty-eight h or 96 h after the training, the rats were placed into a different square chamber in a different room, and 2 min later the same tone was sounded for 5 min without a foot shock. The amount of fear conditioned to the tone was assessed by scoring freezing behavior.

### Auditory threshold test

Rats at 17–21 weeks old were placed in the conditioning chamber for 1 min and were then given 3 s tones (1,000 Hz and 3,000 Hz) of increasing sound intensity. The interval between tones was 10 s. We determined the threshold of sound level required to elicit the orienting reflex to the sound source.

### Electric shock sensitivity test

For the last of the behavioral tests, we measured the sensitivity of rats at 17–21 weeks old to footshock. In this test, each rat was placed in the conditioning chamber and received 1 s shocks of increasing intensity. The interval between shocks was 25 s. The sequence of the current used was as follows: 0.05 mA, 0.08 mA, 0.1 mA, 0.2 mA, 0.3 mA, 0.4 mA, 0.5 mA, 0.6 mA and 0.8 mA. We determined the minimal level of current required to elicit the following stereotypical responses: flinching, running, vocalization, and jumping. These experiments were performed blindly.

### Measurement of ultrasonic vocalizations

A total of 51 rat pups (WT male; 18, WT female; 9, *rSey^2^/+* male; 11, *rSey^2^/+* female; 13) born to 4 dams were tested in USV during the isolation condition on P7. At first, we removed a dam from the home cage. To maintain the pups' body temperature, the home cage was placed on a heat pad maintained at 35°C. A pup was transported in a plastic chamber (170×280×130 cm) with absorbent cotton which was placed in a soundproof box and vocalizations were recorded for 5 min.

USV was recorded with a condenser microphone (CM16/CMPA, Avisoft Bioacoustics, Berlin, Germany) connected to an amplifier/digitizer (Avisoft UltraSoundGate416H, Avisoft Bioacoustics, Berlin, Germany) at a sampling rate of 250 kHz with 125 kHz low-pass filter. The recorded files were transferred to a sound analysis software (SASLab Pro ver. 4.52, Avisoft Bioacoustics, Berlin, Germany) for fast Fourier transform (512 FFT-length, 100% frame size, Hamming window, 50% time window overlap). We analyzed the number of calls, mean duration of one call, latency to start calling and peak frequency of each call.

### Measurement of 5-HT concentration

Blood (2 ml) at 18–20 weeks old was taken from the abdominal vein and drawn into test tubes containing sodium fluoride and EDTA-2Na (Becton Dickinson, Tokyo, Japan), and immediately placed on ice. PPP was separated by centrifugation of the blood samples at 1,500×g for 15 min at 4°C. The resulting PPP (800 µl) was collected and stored at −80°C till assayed. 5-HT analyses of the PPP samples were conducted within 1 week after the experiment. PPP samples (100 µl) were added to 100 µl of 0.5 M perchloric acid and 10 µl of 0.1 mM isoproterenol as an internal standard. After vortex-mixing, the tubes were centrifuged at 2,000×g and 4°C for 15 min, and 40 µl of the resulting supernatant was then injected into the HPLC system, equipped with a reverse-phase chromatographic column (Eicompak, SC-5ODS, 3 mm diameter×150 mm, Eicom, Kyoto, Japan). The applied potential was +400 mV versus the Ag/AgCl electrode. The mobile phase consisted of a 0.1 M sodium phosphate buffer (pH 6.0)-methanol (8 : 2, v/v) containing 400 mg/L sodium 1-octanesulfonate and 50 mg/L EDTA-2Na. The flow rate was set at 0.5 ml/min and the column temperature was maintained at 25°C. Compounds were detected using an electrochemical detector (Eicom ECD-300, Eicom, Kyoto, Japan) and quantitated by comparison of peaks area to that of the internal standard [76].

Hippocampus samples at 12 weeks old were homogenized in 5 volumes of 0.2 M perchloric acid containing 0.1 M EDTA and the proper concentration of isoproterenol. After centrifugation of the homogenates, the pH of the supernatant was adjusted to 3.0 by adding 1 M sodium acetate. The mobile phase was 0.1 M sodium citrate, 0.1 M citric acid, 0.5 mM sodium octanesulfonate, 0.15 mM EDTA, and 12% methanol, pH 3.5 [77].

### Measurement of auditory startle and prepulse inhibition of acoustic startle

Rats at 12–17 weeks old were tested in a startle chamber (SR-Lab Systems, San Diego Instruments, San Diego, CA) positioned within a soundproof cabinet in a sound-attenuating room according to the method previously described [78], [79]. A constant background noise of 65 dB was presented throughout the test. To measure PPI scores rats were given 20-ms-long 68, 71, or 77 dB prepulse that preceded the 120 dB pulse (40 ms width) by 100 ms (pp68, pp71, pp77). Percent PPI of a startle response was calculated: 100 – [(startle response on acoustic prepulse and startle stimulus trials/startle response alone trials) ×100].

### Treatment with clozapine

Rats at 12–17 weeks old were injected intraperitoneally with 0.5 mg/ml of clozapine (Sigma) to make the final dose at 1.5 mg/kg body weight or with the similar amount of saline (control) according to the body weight. Both rats were analyzed for the same behavior tests as described above.

### Data analysis

Data were analyzed by Student's *t* test, two-way ANOVA or two-way ANOVA with repeated measures followed by Bonferroni post hoc where appropriate, using SPSS version 16 software (SPSS Inc., Chicago, IL, USA). A P value below 0.05 was considered to be significant. All values in the text and figure legends were expressed as means plus/minus standard error of the mean (SEM), and n is the number of rats tested except for social interaction test where n indicates the number of pairs of rats examined.

## References

[1] Levy SE, Mandell DS, Schultz RT (2009). Autism.. Lancet.

[2] Steffenburg S, Gillberg C, Hellgren L, Andersson L, Gillberg IC (1989). A twin study of autism in Denmark, Finland, Iceland, Norway and Sweden.. J Child Psychol Psychiatry.

[3] Geschwind DH (2009). Advances in autism.. Annu Rev Med.

[4] Association AP (2000). Diagnostic and Statistical Manual of Mental Disorders.. edn.

[5] Ospina MB, Krebs Seida J, Clark B, Karkhaneh M, Hartling L (2008). Behavioural and developmental interventions for autism spectrum disorder: a clinical systematic review.. PLoS One.

[6] Hughes JR (2009). Update on autism: a review of 1300 reports published in 2008.. Epilepsy Behav.

[7] Senju A, Johnson MH (2009). Atypical eye contact in autism: models, mechanisms and development.. Neurosci Biobehav Rev.

[8] Ronald A, Happe F, Bolton P, Butcher LM, Price TS (2006). Genetic heterogeneity between the three components of the autism spectrum: a twin study.. J Am Acad Child Adolesc Psychiatry.

[9] Dworzynski K, Happe F, Bolton P, Ronald A (2009). Relationship between symptom domains in autism spectrum disorders: a population based twin study.. J Autism Dev Disord.

[10] Bailey A, Le Couteur A, Gottesman I, Bolton P, Simonoff E (1995). Autism as a strongly genetic disorder: evidence from a British twin study.. Psychol Med.

[11] Freitag CM, Staal W, Klauck SM, Duketis E, Waltes R (2010). Genetics of autistic disorders: review and clinical implications.. Eur Child Adolesc Psychiatry.

[12] Becker KG (2004). The common variants/multiple disease hypothesis of common complex genetic disorders.. Med Hypotheses.

[13] Bill BR, Geschwind DH (2009). Genetic advances in autism: heterogeneity and convergence on shared pathways.. Curr Opin Genet Dev.

[14] Walther C, Gruss P (1991). Pax-6, a murine paired box gene, is expressed in the developing CNS.. Development.

[15] Stoykova A, Gruss P (1994). Roles of Pax-genes in developing and adult brain as suggested by expression patterns.. J Neurosci.

[16] Nakatomi H, Kuriu T, Okabe S, Yamamoto S, Hatano O (2002). Regeneration of hippocampal pyramidal neurons after ischemic brain injury by recruitment of endogenous neural progenitors.. Cell.

[17] Sakurai K, Osumi N (2008). The neurogenesis-controlling factor, Pax6, inhibits proliferation and promotes maturation in murine astrocytes.. J Neurosci.

[18] Ton CC, Hirvonen H, Miwa H, Weil MM, Monaghan P (1991). Positional cloning and characterization of a paired box- and homeobox-containing gene from the aniridia region.. Cell.

[19] Hanson I, Van Heyningen V (1995). Pax6: more than meets the eye.. Trends Genet.

[20] Fischbach BV, Trout KL, Lewis J, Luis CA, Sika M (2005). WAGR syndrome: a clinical review of 54 cases.. Pediatrics.

[21] Xu S, Han JC, Morales A, Menzie CM, Williams K (2008). Characterization of 11p14-p12 deletion in WAGR syndrome by array CGH for identifying genes contributing to mental retardation and autism.. Cytogenet Genome Res.

[22] Malandrini A, Mari F, Palmeri S, Gambelli S, Berti G (2001). PAX6 mutation in a family with aniridia, congenital ptosis, and mental retardation.. Clin Genet.

[23] Davis LK, Meyer KJ, Rudd DS, Librant AL, Epping EA (2008). Pax6 3′ deletion results in aniridia, autism and mental retardation.. Hum Genet.

[24] Graziano C, D'Elia AV, Mazzanti L, Moscano F, Guidelli Guidi S (2007). A de novo nonsense mutation of PAX6 gene in a patient with aniridia, ataxia, and mental retardation.. Am J Med Genet A.

[25] Szatmari P, Paterson AD, Zwaigenbaum L, Roberts W, Brian J (2007). Mapping autism risk loci using genetic linkage and chromosomal rearrangements.. Nat Genet.

[26] Maekawa M, Iwayama Y, Nakamura K, Sato M, Toyota T (2009). A novel missense mutation (Leu46Val) of PAX6 found in an autistic patient.. Neurosci Lett.

[27] Osumi N, Hirota A, Ohuchi H, Nakafuku M, Iimura T (1997). Pax-6 is involved in the specification of hindbrain motor neuron subtype.. Development.

[28] Matsuo T, Osumi-Yamashita N, Noji S, Ohuchi H, Koyama E (1993). A mutation in the Pax-6 gene in rat small eye is associated with impaired migration of midbrain crest cells.. Nat Genet.

[29] Becker A, Grecksch G, Bernstein HG, Hollt V, Bogerts B (1999). Social behaviour in rats lesioned with ibotenic acid in the hippocampus: quantitative and qualitative analysis.. Psychopharmacology (Berl).

[30] Ikegami S, Inokuchi K (2000). Antisense DNA against calcineurin facilitates memory in contextual fear conditioning by lowering the threshold for hippocampal long-term potentiation induction.. Neuroscience.

[31] Bouwknecht JA, van der Gugten J, Groenink L, Olivier B, Paylor RE (2004). Effects of repeated testing in two inbred strains on flesinoxan dose-response curves in three mouse models for anxiety.. Eur J Pharmacol.

[32] Wells CE, Krikke B, Saunders J, Whittington A, Lever C (2009). Changes to open field surfaces typically used to elicit hippocampal remapping elicit graded exploratory responses.. Behav Brain Res.

[33] Costall B, Coughlan J, Horovitz ZP, Kelly ME, Naylor RJ (1989). The effects of ACE inhibitors captopril and SQ29,852 in rodent tests of cognition.. Pharmacol Biochem Behav.

[34] Kanakubo S, Nomura T, Yamamura K, Miyazaki J, Tamai M (2006). Abnormal migration and distribution of neural crest cells in Pax6 heterozygous mutant eye, a model for human eye diseases.. Genes Cells.

[35] Porsolt RD, Anton G, Blavet N, Jalfre M (1978). Behavioural despair in rats: a new model sensitive to antidepressant treatments.. Eur J Pharmacol.

[36] Phillips RG, LeDoux JE (1992). Differential contribution of amygdala and hippocampus to cued and contextual fear conditioning.. Behav Neurosci.

[37] Branchi I, Santucci D, Alleva E (2001). Ultrasonic vocalisation emitted by infant rodents: a tool for assessment of neurobehavioural development.. Behav Brain Res.

[38] Anderson GM (2002). Genetics of childhood disorders: XLV. Autism, part 4: serotonin in autism.. J Am Acad Child Adolesc Psychiatry.

[39] Anderson GM, Horne WC, Chatterjee D, Cohen DJ (1990). The hyperserotonemia of autism.. Ann N Y Acad Sci.

[40] Huang CH, Santangelo SL (2008). Autism and serotonin transporter gene polymorphisms: a systematic review and meta-analysis.. Am J Med Genet B Neuropsychiatr Genet.

[41] Maekawa M, Takashima N, Matsumata M, Ikegami S, Kontani M (2009). Arachidonic acid drives postnatal neurogenesis and elicits a beneficial effect on prepulse inhibition, a biological trait of psychiatric illnesses.. PLoS One.

[42] Brieden T, Ujeyl M, Naber D (2002). Psychopharmacological treatment of aggression in schizophrenic patients.. Pharmacopsychiatry.

[43] Osumi N (2001). The role of Pax6 in brain patterning.. Tohoku J Exp Med.

[44] Glaser T, Jepeal L, Edwards JG, Young SR, Favor J (1994). PAX6 gene dosage effect in a family with congenital cataracts, aniridia, anophthalmia and central nervous system defects.. Nat Genet.

[45] Schmahl W, Knoedlseder M, Favor J, Davidson D (1993). Defects of neuronal migration and the pathogenesis of cortical malformations are associated with Small eye (Sey) in the mouse, a point mutation at the Pax-6-locus.. Acta Neuropathol.

[46] Maekawa M, Takashima N, Arai Y, Nomura T, Inokuchi K (2005). Pax6 is required for production and maintenance of progenitor cells in postnatal hippocampal neurogenesis.. Genes Cells.

[47] Haba H, Nomura T, Suto F, Osumi N (2009). Subtype-specific reduction of olfactory bulb interneurons in Pax6 heterozygous mutant mice.. Neurosci Res.

[48] Sisodiya SM, Free SL, Williamson KA, Mitchell TN, Willis C (2001). PAX6 haploinsufficiency causes cerebral malformation and olfactory dysfunction in humans.. Nat Genet.

[49] Free SL, Mitchell TN, Williamson KA, Churchill AJ, Shorvon SD (2003). Quantitative MR image analysis in subjects with defects in the PAX6 gene.. Neuroimage.

[50] Mitchell TN, Free SL, Williamson KA, Stevens JM, Churchill AJ (2003). Polymicrogyria and absence of pineal gland due to PAX6 mutation.. Ann Neurol.

[51] Hardan AY, Pabalan M, Gupta N, Bansal R, Melhem NM (2009). Corpus callosum volume in children with autism.. Psychiatry Res.

[52] Nishina S, Kohsaka S, Yamaguchi Y, Handa H, Kawakami A (1999). PAX6 expression in the developing human eye.. Br J Ophthalmol.

[53] Tuoc TC, Radyushkin K, Tonchev AB, Pinon MC, Ashery-Padan R (2009). Selective cortical layering abnormalities and behavioral deficits in cortex-specific Pax6 knock-out mice.. J Neurosci.

[54] Guo H, Hong S, Jin XL, Chen RS, Avasthi PP (2000). Specificity and efficiency of Cre-mediated recombination in Emx1-Cre knock-in mice.. Biochem Biophys Res Commun.

[55] Perry W, Minassian A, Lopez B, Maron L, Lincoln A (2007). Sensorimotor gating deficits in adults with autism.. Biol Psychiatry.

[56] McAlonan GM, Daly E, Kumari V, Critchley HD, van Amelsvoort T (2002). Brain anatomy and sensorimotor gating in Asperger's syndrome.. Brain.

[57] Klin A, Saulnier CA, Sparrow SS, Cicchetti DV, Volkmar FR (2007). Social and communication abilities and disabilities in higher functioning individuals with autism spectrum disorders: the Vineland and the ADOS.. J Autism Dev Disord.

[58] Frith U (1993). Autism.. Sci Am.

[59] Jamain S, Radyushkin K, Hammerschmidt K, Granon S, Boretius S (2008). Reduced social interaction and ultrasonic communication in a mouse model of monogenic heritable autism.. Proc Natl Acad Sci U S A.

[60] Shu W, Cho JY, Jiang Y, Zhang M, Weisz D (2005). Altered ultrasonic vocalization in mice with a disruption in the Foxp2 gene.. Proc Natl Acad Sci U S A.

[61] Young DM, Schenk AK, Yang SB, Jan YN, Jan LY (2010). Altered ultrasonic vocalizations in a tuberous sclerosis mouse model of autism.. Proc Natl Acad Sci U S A.

[62] Wing L (1981). Sex ratios in early childhood autism and related conditions.. Psychiatry Res.

[63] Hartley SL, Sikora DM (2009). Sex Differences in Autism Spectrum Disorder: An Examination of Developmental Functioning, Autistic Symptoms, and Coexisting Behavior Problems in Toddlers.. J Autism Dev Disord.

[64] McEuen JG, Semsar KA, Lim MA, Bale TL (2009). Influence of sex and corticotropin-releasing factor pathways as determinants in serotonin sensitivity.. Endocrinology.

[65] Shors TJ, Chua C, Falduto J (2001). Sex differences and opposite effects of stress on dendritic spine density in the male versus female hippocampus.. J Neurosci.

[66] Wellmann K, Lewis B, Barron S (2010). Agmatine reduces ultrasonic vocalization deficits in female rat pups exposed neonatally to ethanol.. Neurotoxicol Teratol.

[67] Cook EH, Leventhal BL (1996). The serotonin system in autism.. Curr Opin Pediatr.

[68] Spivak B, Golubchik P, Mozes T, Vered Y, Nechmad A (2004). Low platelet-poor plasma levels of serotonin in adult autistic patients.. Neuropsychobiology.

[69] Rigdon GC, Weatherspoon JK (1992). 5-Hydroxytryptamine 1a receptor agonists block prepulse inhibition of acoustic startle reflex.. J Pharmacol Exp Ther.

[70] Braff DL, Geyer MA, Swerdlow NR (2001). Human studies of prepulse inhibition of startle: normal subjects, patient groups, and pharmacological studies.. Psychopharmacology (Berl).

[71] Van Steenwinckel J, Noghero A, Thibault K, Brisorgueil MJ, Fischer J (2009). The 5-HT2A receptor is mainly expressed in nociceptive sensory neurons in rat lumbar dorsal root ganglia.. Neuroscience.

[72] Brauer D, Strobel A, Hensch T, Diers K, Lesch KP (2009). Genetic variation of serotonin receptor function affects prepulse inhibition of the startle.. J Neural Transm.

[73] Arranz M, Collier D, Sodhi M, Ball D, Roberts G (1995). Association between clozapine response and allelic variation in 5-HT2A receptor gene.. Lancet.

[74] Burnet PW, Chen CP, McGowan S, Franklin M, Harrison PJ (1996). The effects of clozapine and haloperidol on serotonin-1A, -2A and -2C receptor gene expression and serotonin metabolism in the rat forebrain.. Neuroscience.

[75] Shinohara T, Tomizuka K, Miyabara S, Takehara S, Kazuki Y (2001). Mice containing a human chromosome 21 model behavioral impairment and cardiac anomalies of Down's syndrome.. Hum Mol Genet.

[76] Danaceau JP, Anderson GM, McMahon WM, Crouch DJ (2003). A liquid chromatographic-tandem mass spectrometric method for the analysis of serotonin and related indoles in human whole blood.. J Anal Toxicol.

[77] Sano H, Yasoshima Y, Matsushita N, Kaneko T, Kohno K (2003). Conditional ablation of striatal neuronal types containing dopamine D2 receptor disturbs coordination of basal ganglia function.. J Neurosci.

[78] Swerdlow NR, Martinez ZA, Hanlon FM, Platten A, Farid M (2000). Toward understanding the biology of a complex phenotype: rat strain and substrain differences in the sensorimotor gating-disruptive effects of dopamine agonists.. J Neurosci.

[79] Kew JN, Koester A, Moreau JL, Jenck F, Ouagazzal AM (2000). Functional consequences of reduction in NMDA receptor glycine affinity in mice carrying targeted point mutations in the glycine binding site.. J Neurosci.

